# Personality trait by intelligence interaction effects on grades tend to be synergistic

**DOI:** 10.1186/s40359-021-00708-1

**Published:** 2021-12-28

**Authors:** Kimmo Sorjonen, Alma Sörberg Wallin, Daniel Falkstedt, Bo Melin

**Affiliations:** 1 Division of Psychology, Department of Clinical Neuroscience, Karolinska Institutet, 171 77 Stockholm, Sweden; 2 Department of Global Public Health, Karolinska Institutet, Stockholm, Sweden; 3 Institute of Environmental Medicine, Karolinska Institutet, Stockholm, Sweden

**Keywords:** Academic achievement, Intelligence, Interaction effect, Personality traits, Reliability, Reversed causality, Synergistic

## Abstract

**Background:**

Earlier research has identified both synergistic and compensatory personality traits by intelligence interaction effects on academic performance.

**Methods:**

The present study employed data on intelligence, personality traits, and academic performance in the 1997 National Longitudinal Survey of Youth (NLSY97, *N* = 8984).

**Results:**

Some intelligence by personality trait interaction effects, mainly involving indicators of dependability, on high school grades were identified. The interaction effects tended to be synergistic, meaning that the association between the trait and grades tended to strengthen with increased intelligence. A positive association between intelligence and the reliability in the measurement of a dependability composite score accounted for a substantial portion of the synergistic dependability by intelligence interaction effect on academic performance.

**Conclusions:**

Personality trait by intelligence interaction effects on academic performance tend to be synergistic and may, at least to some degree, be due to a positive association between intelligence and reliability in the measurement of personality traits.

## Introduction

Studies have identified personality trait by intelligence interaction effects on academic performance. More often than not, these interaction effects seem to be synergistic, i.e. the association between the personality trait and academic performance strengthens with increased intelligence. For example, among students, an increase in intelligence has been found to strengthen the positive association between grades and conscientiousness [1–3]. Lozano et al. [4] observed that impulsivity was more strongly negatively associated with grades among more intelligent compared to less intelligent students. Furthermore, in a study among mainly Hispanic STEM (Science, Technology, Engineering, and Mathematics) students, an increase in intelligence strengthened the negative association between agreeableness and grades [5]. Heaven and Ciarrochi [6] observed a stronger association between openness/intellect and grades among students with higher compared to those with lower intelligence. However, compensatory interaction effects, where the association between the trait and academic outcomes weaken with increased intelligence, have also been reported. For example, an increase in intelligence seems to weaken the positive association between grades and need for cognition [7]. In contradiction to the finding by Heaven and Ciarrochi [6] mentioned above, Zhang and Ziegler [8] found that the positive association between openness and grades in a sample of students weakened with increased intelligence. An increase in fluid intelligence has also been found to weaken the positive association between openness and crystallized intelligence [9].

**Fig. 1:**
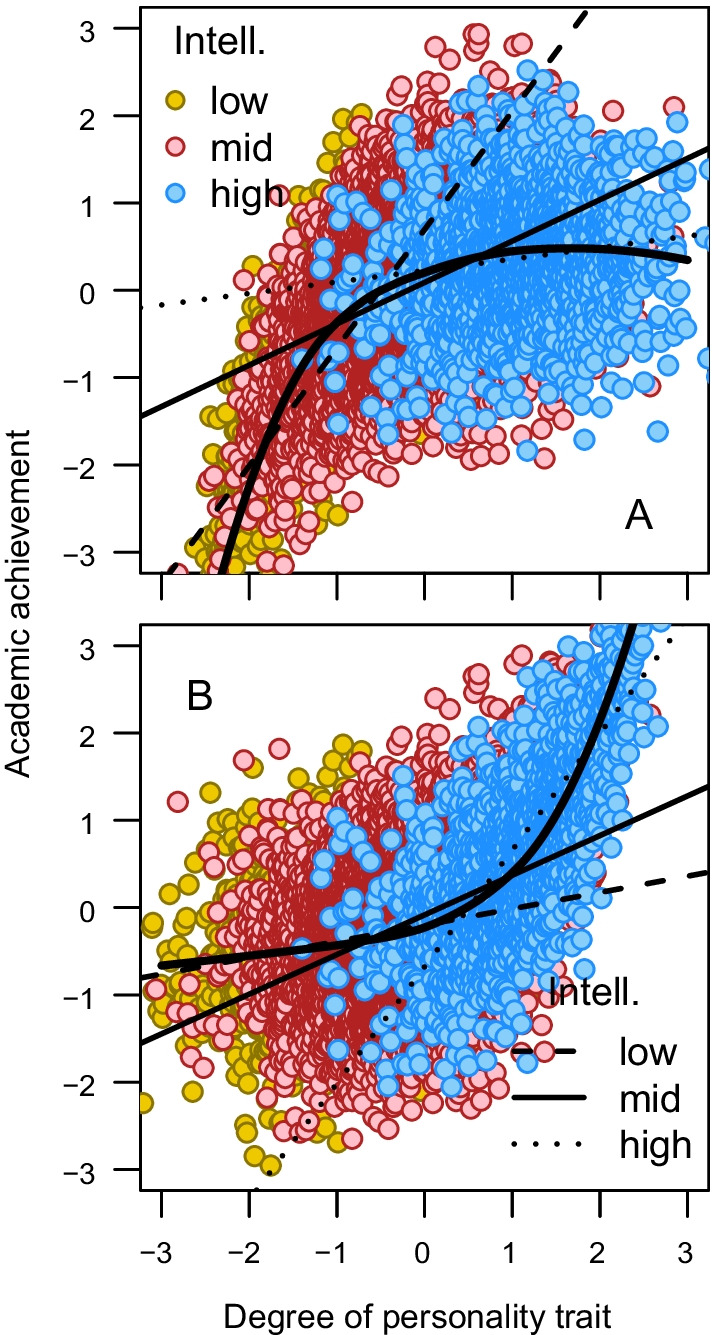
A compensatory (**A**) and a synergistic (**B**) intelligence by personality trait interaction effect on academic achievement that is due to a concave (**A**) and a convex (**B**) association between personality trait and academic achievement, respectively, in combination with a positive association between intelligence and the personality trait. Simulated data

However, observed interaction effects should not always be taken at face value. In his pivotal paper, Ganzach [10] made the simple, but eloquent, argument that if two predictors, X_1_ and X_2_, are correlated, the X_1_ × X_2_ interaction term will tend to be correlated with the quadratic terms X_1_^2^ and X_2_^2^ and, consequently, an identified interaction effect on the outcome Y may be due to a quadratic association between Y and X_1_ or X_2_ or, vice versa, an identified quadratic association between Y and X_1_ or X_2_ may be due to an interaction effect (Fig. 1). Therefore, it is advisable to adjust for possible quadratic associations when claiming that X_1_ and X_2_ interact in their effect on Y, and, vice versa, to adjust for the interaction if claiming a quadratic association.

The objective of the present study was to: (1) Analyze if there are intelligence by personality trait interaction effects on academic achievement, operationalized as grade point average and highest degree achieved, in the 1997 National Longitudinal Study of Youth (NLSY97) dataset; (2) Analyze if interaction effects remain significant when adjusting for possibly quadratic associations between personality trait/intelligence and academic achievement; (3) Evaluate if adjusted interaction effects tend to be synergistic or compensatory.

## Method

### Respondents

Data were collected from 8984 US respondents (4385 women and 4599 men), born between 1980 and 1984, who took part in the 1997 National Longitudinal Survey of Youth (NLSY97).

### Measurements

Most respondents (complete data available for 7008 individuals) took 12 Armed Services Vocational Aptitude Battery (ASVAB) tests in 1997–1998: (1) general science; (2) arithmetic reasoning; (3) word knowledge; (4) paragraph comprehension; (5) numerical operations; (6) coding speed; (7) auto information; (8) shop information; (9) mathematical knowledge; (10) mechanical comprehension; (11) electronics information; (12) assembling objects.

On later occasions, the respondents rated to what degree they felt that a number of personality trait items were descriptive of themselves: (1) disorganized, (2) conscientious, (3) undependable, (4) thorough, (5) agreeable, (6) difficult, (7) stubborn, (8) trustful, (9) extraverted/enthusiastic, (10) critical/quarrelsome, (11) dependable/self-disciplined, (12) anxious/easily upset, (13) open/complex, (14) reserved/quiet, (15) sympathetic/warm, (16) disorganized/careless, (17) calm/emotionally stable, (18) conventional/uncreative. Items 1–8 were rated in 2002 on a scale from 1 to 5 (*n* between 4857 and 4875) while items 9–18 were rated in 2008 on a scale from 1 to 7 (*n* between 7195 and 7457). The different response scales for items 1–8 and 9–18 were handled by standardizing the scores before calculations.

Based on item content, the 18 personality trait items were initially grouped into the following three composite scores: (a) Dependable, with the items (see above) 1 (reversed), 2, 3 (reversed), 4, 11, and 16 (reversed); (b) Agreeable, with the items 5, 6 (reversed), 7 (reversed), 8, 10 (reversed), and 15; (c) Open, with the items 9, 12 (reversed), 13, 14 (reversed), 17, and 18 (reversed). As item 2 (conscientious) had a very low factor loading (0.167) and decreased the measured homogeneity of the composite, it was removed. This increased the fit of the factor structure (Akaike Information Criterion (AIC) decreased from 313,257 to 299,545). The composites dependable and agreeable correspond quite closely to the Big Five personality traits Conscientiousness and Agreeableness, respectively [11]. However, we chose to name the first composite dependable rather than conscientious as the conscientious item was removed from the composite. The open composite could be seen to correspond to a combination of the Big Five personality traits Openness to Experience, Extraversion, and low Neuroticism. We are aware that this third composite is a bit of a hodgepodge. However, the main objective of the present study was to evaluate if intelligence by personality trait interaction effects on academic achievement tend to be synergistic or compensatory, and this objective should be achievable even if not all of the used personality trait measures are perfectly orderly. In general terms, to estimate if the A by B interaction effect on C is synergistic or compensatory is possible even if it is not as clear what B measures. The homogeneity (as measured by coefficient Omega) was 0.54, 0.55, and 0.52 for the composites dependable, agreeable, and open, respectively.

Credit weighted overall grade point average (GPA) was available, as transcript survey data from high schools, for 6004 respondents. Moreover, in 2017 respondents were asked about their highest academic degree received, with the values: (0) None, *n* = 515, (1) General educational development, *n* = 862, (2) High school diploma, *n* = 2692, (3) Associate/junior college, *n* = 598, (4) Bachelor’s degree, *n* = 1352, (5) Master’s degree, *n* = 540, (6) Professional degree/PhD, *n* = 149. Data on degree was available for 6708 respondents.

### Statistical analyses

Factor scores on the first unrotated factor in an analysis of all 12 ASVAB tests was used as an estimate of the respondents’ general intelligence. The scores on each personality trait item as well as the three composites were standardized and the correlation with intelligence and grades/degree as well as the item by intelligence interaction effect on grades/degree were calculated. The interaction effects were estimated both without and with adjustment for possible quadratic associations. In the latter case the predicted grade/degree is given by:

1 $$E\left| {gr/de} \right| = b_{0} + b_{1} \times intell + b_{2} \times intell^{2} + b_{3} \times trait + b_{4} \times trait^{2} + b_{5} \times intell \times trait$$

If the personality trait by intelligence interaction effect on grades/degree tends to be synergistic or complementary, we should see a positive or a negative association between the item-grades/degree correlation and the coefficient for the item by intelligence interaction effect, respectively. The present study included 195 tests of significance, and if wishing to be cautious not to conduct type 1-errors, a conservative Bonferroni correction could be used to set the significance level at 0.05/195 = 0.000256. Analyses were conducted with R 4.0.2 statistical software [12] employing the psych [13] and lavaan [14] packages. Code and dataset are available at https://osf.io/2fsca/.

## Results

Intelligence had a positive association both with grade point average (β = 0.459, *p* < 0.001) and with highest degree achieved (β = 0.506, *p* < 0.001). A quadratic intelligence term was significant in the model predicting grades (β = 0.039, *p* < 0.001) but it only contributed to increase *R*-squared from 0.204 to 0.206. A quadratic intelligence term had no significant association with highest degree (*p* = 0.922).

Associations involving the 18 personality trait items and the three composite scores can be seen in Table 1. It can be noted that (a) The personality traits tend to have only weak linear associations with intelligence (column 1), grades (column 2), and degree (column 6), with conscientious (row 2) and the open composite score (row 21) having the strongest positive associations and undependable (row 3) and anxious (row 12) the strongest negative associations; (b) Only conscientious has a significant positive quadratic association with grades (but not with degree) while several other personality trait items—including stubborn, anxious, and reserved—have a negative quadratic associations both with grades and with degree. This suggests that with a further increase beyond a certain level, the negative association between these traits and grades/degree strengthen with increasing speed; (c) The personality trait by intelligence interaction effects do not change much when adjusting for quadratic terms (compare columns 4 and 5, and 8 and 9, respectively); (d) Among the interaction effects we can note that the negative associations between grades and disorganized, critical, and careless strengthen with increasing intelligence as does the positive association between grades and the dependable composite score.

**Table 1:** Correlations and standardized regression effects between study variables

Personality item		Grade point average				Highest degree achieved			
	1. *r*(iq)^a^	2. *r*(gr)^b^	3. *b*(pe^2^)^c^	4. Int-C^d^	5. Int-A^e^	6. *r*(de)^b^	7. *b*(pe^2^)^c^	8. Int-C^d^	9 Int-A^e^
1. Disorganized^f^	0.032*	− 0.074**	0.005	− 0.069**	− 0.072**	− 0.084**	0.005	− 0.028	− 0.028
2. Conscientious	0.188**	0.167**	0.041*	0.043*	0.035	0.139**	0.016	0.040*	0.043*
3. Undependable^f^	− 0.128**	− 0.125**	0.027	− 0.068*	− 0.062*	− 0.159**	0.018	− 0.009	− 0.005
4. Thorough^f^	0.080**	0.090**	− 0.021	0.037*	0.033	0.102**	− 0.038*	0.002	0.002
5. Agreeable^g^	0.028	0.088**	− 0.022	0.026	0.024	0.097**	− 0.023	0.021	0.023
6. Difficult^g^	− 0.085**	− 0.114**	− 0.048*	− 0.025	− 0.024	− 0.134**	− 0.052**	− 0.004	− 0.004
7. Stubborn^g^	0.080**	− 0.001	− 0.065**	0.008	0.003	− 0.031	− 0.080**	− 0.018	− 0.016
8. Trustful^g^	0.059**	0.057*	− 0.012	0.032	0.032	0.067**	− 0.007	− 0.001	− 0.001
9. Extraverted^h^	0.009	0.043*	0.000	− 0.005	− 0.003	0.072**	− 0.009	− 0.017	− 0.018
10. Critical^g^	− 0.043*	− 0.054**	− 0.007	− 0.052**	− 0.052**	− 0.057**	− 0.030*	− 0.027*	− 0.028*
11. Dependable^f^	− 0.001	0.072**	− 0.021*	0.041*	0.039*	0.076**	− 0.045**	0.036*	0.042*
12. Anxious^h^	− 0.199**	− 0.109**	− 0.070**	− 0.005	0.001	− 0.169**	− 0.100**	0.021	0.010
13. Open^h^	0.018	− 0.003	− 0.052**	− 0.003	0.002	0.056**	− 0.069**	0.004	0.010
14. Reserved^h^	− 0.122**	− 0.062**	− 0.053*	− 0.021	− 0.019	− 0.085**	− 0.098**	0.016	0.011
15. Sympathetic^g^	0.050**	0.069**	− 0.016	0.039*	0.037*	0.093**	− 0.049**	0.014	0.016
16. Careless^f^	0.044*	− 0.065**	− 0.048*	− 0.048*	− 0.051**	− 0.067**	− 0.082**	− 0.028*	− 0.029*
17. Calm^h^	0.033*	0.045*	− 0.029*	0.014	0.012	0.087**	− 0.050**	0.015	0.017
18. Conventional^h^	− 0.077**	− 0.007	− 0.060**	− 0.001	0.003	− 0.034*	− 0.070**	0.012	0.012
*Composite*									
19. Dependable	0.012	0.116**	− 0.021*	0.066**	0.066**	0.125**	− 0.022*	0.032*	0.033*
20. Agreeable	0.057**	0.097**	− 0.002	0.041*	0.042*	0.119**	0.001	0.014	0.014
21. Open	0.142**	0.082**	− 0.020*	0.010	0.004	0.157**	− 0.026*	− 0.022	− 0.023

^a^Correlation with intelligence, ^b^correlation with grades/degree, ^c^quadratic term for the association with grades/degree, ^d^crude intelligence by personality trait interaction effect on grades/degree, not adjusting for quadratic terms, ^e^intelligence by personality trait interaction effect on grades/degree, adjusted for quadratic terms, ^f^included in the Dependable composite, ^g^included in the Agreeable composite, ^h^included in the Open composite, *p < 0.05, **p < 0.000256

**Fig. 2:**
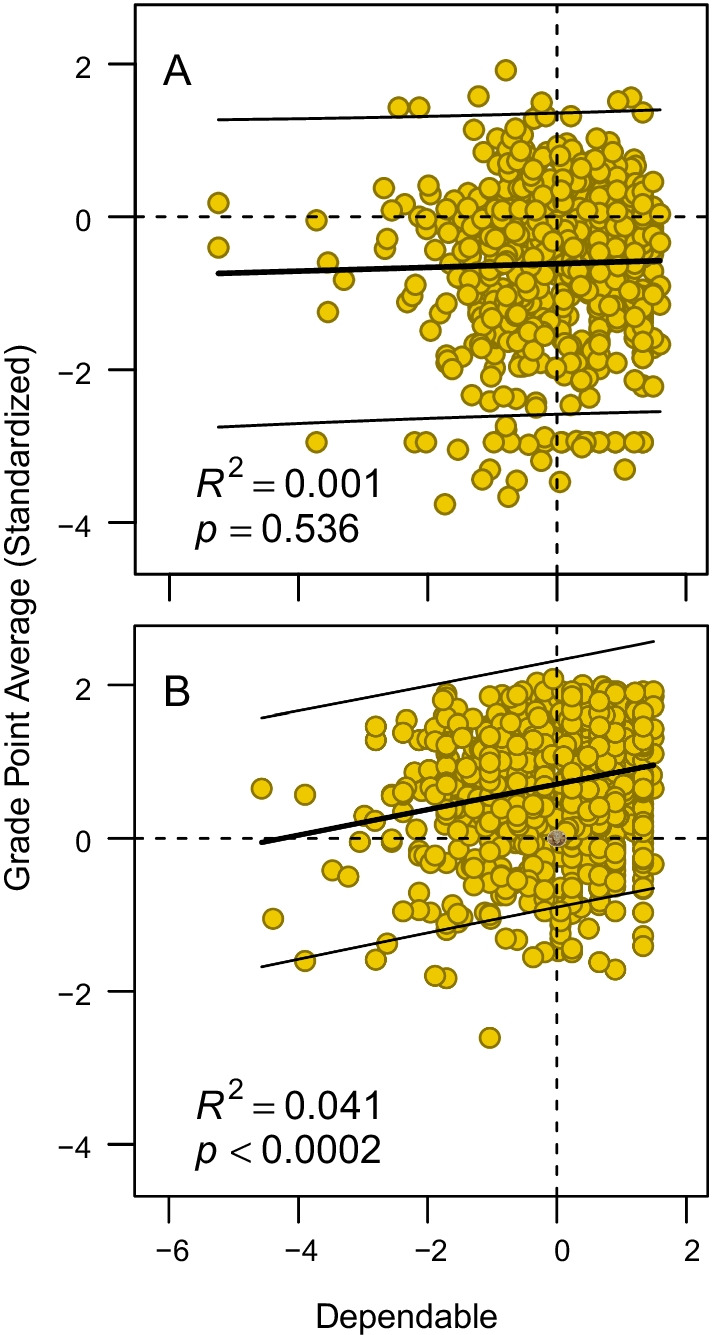
Grade point average as a function of dependable composite score, separately for those with low (≤ − 1 standardized score) (**A**) and high (≥ 1) (**B**) intelligence. The thinner solid lines indicate the 95% prediction interval

A closer look at the intelligence by dependable composite score interaction effect on grades indicates that the weaker (actually non-existent) association among those with low intelligence (≤ − 1 standardized score) may, at least to some degree, be due to some individuals rating themselves high on the personality trait but still receiving very low grades (Fig. 2, panel A). This combination of high self-rated dependability and low grades is not seen to the same degree among those with high (*Z* ≥ 1) intelligence (Fig. 2, panel B). It can, furthermore, be noted that the reliability in the measurement of the dependable composite score was lower among those with low compared to those with high intelligence, with coefficient omega equal to 0.47 and 0.66, respectively. A similar difference in reliability was seen on the agreeable (omega = 0.44 and 0.68, respectively) but not on the open (omega = 0.52 for both groups) composite scores.

**Fig. 3:**
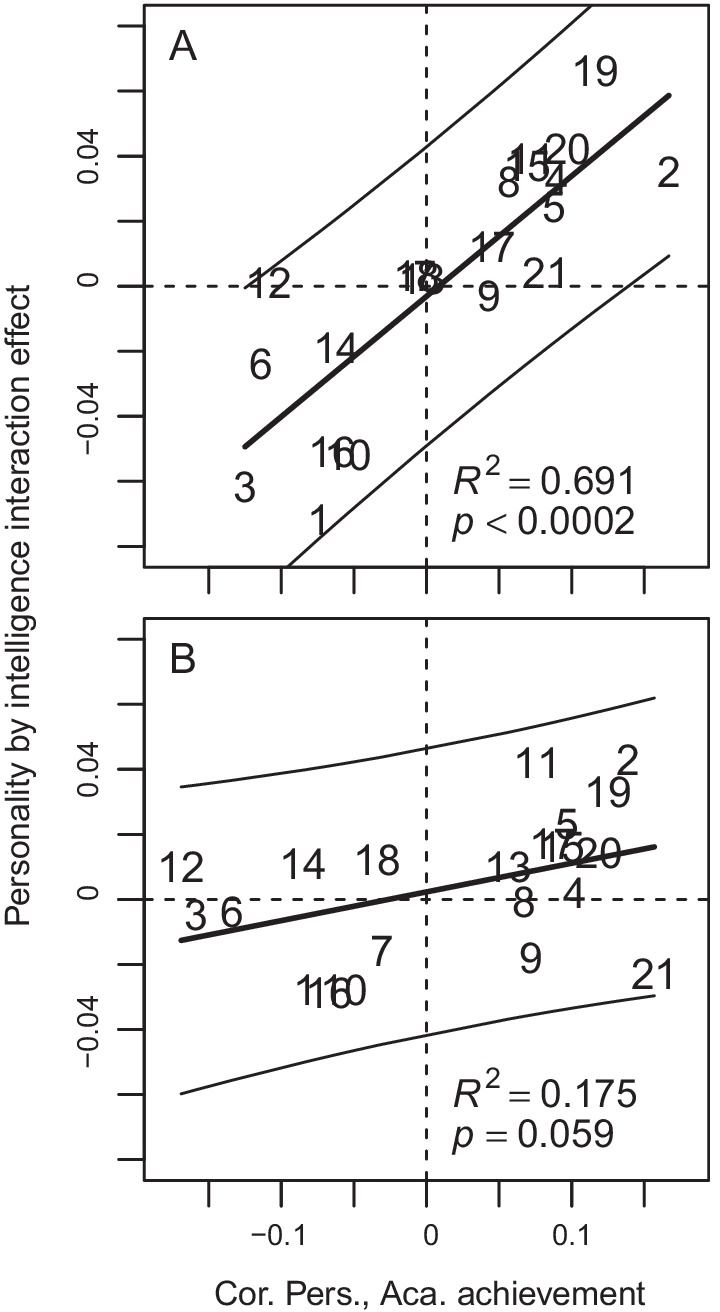
The personality trait by intelligence interaction effect on grade point average (**A**) and highest degree achieved (**B**) as a function of the correlation between the personality trait and grades/degree. The numbers correspond to rows in Table 1, where the interaction effects can be found in columns 5 and 9 and the correlations in columns 2 and 6, respectively. The thinner solid lines indicate the 95% prediction interval

A positive association between the correlation between the personality items/composites and academic achievement (columns 2 and 6 in Table 1 for grades and for degree, respectively) and the personality trait by intelligence interaction term (columns 5 and 9 in Table 1) indicates that the interaction effects tend to be synergistic, at least on grades (Fig. 3). This means that the association between personality traits and grades, positive or negative, tend to strengthen with an increase in intelligence.

The observed tendency for synergistic personality trait by intelligence interaction effects on academic achievement may, at least partly, be due to the observed difference in reliability in the measurement of personality traits between those with high and low intelligence, respectively. Therefore, additional analyses were conducted. Based on their intelligence, the respondents were included in one or several of 901 subgroups with a range of 0.5 in standardized intelligence (i.e. with overlap between the subgroups and most respondents included in more than one subgroup). In each subgroup we calculated: (1) Mean intelligence; (2) The homogeneity (coefficient Omega) of the dependable composite; (3) The regression effects of the dependable composite on grades and degree, respectively. These analyses were restricted to the dependable composite as the two other composites, agreeable and open, did not interact synergistically with intelligence in their effect on academic achievement.

**Fig. 4:**
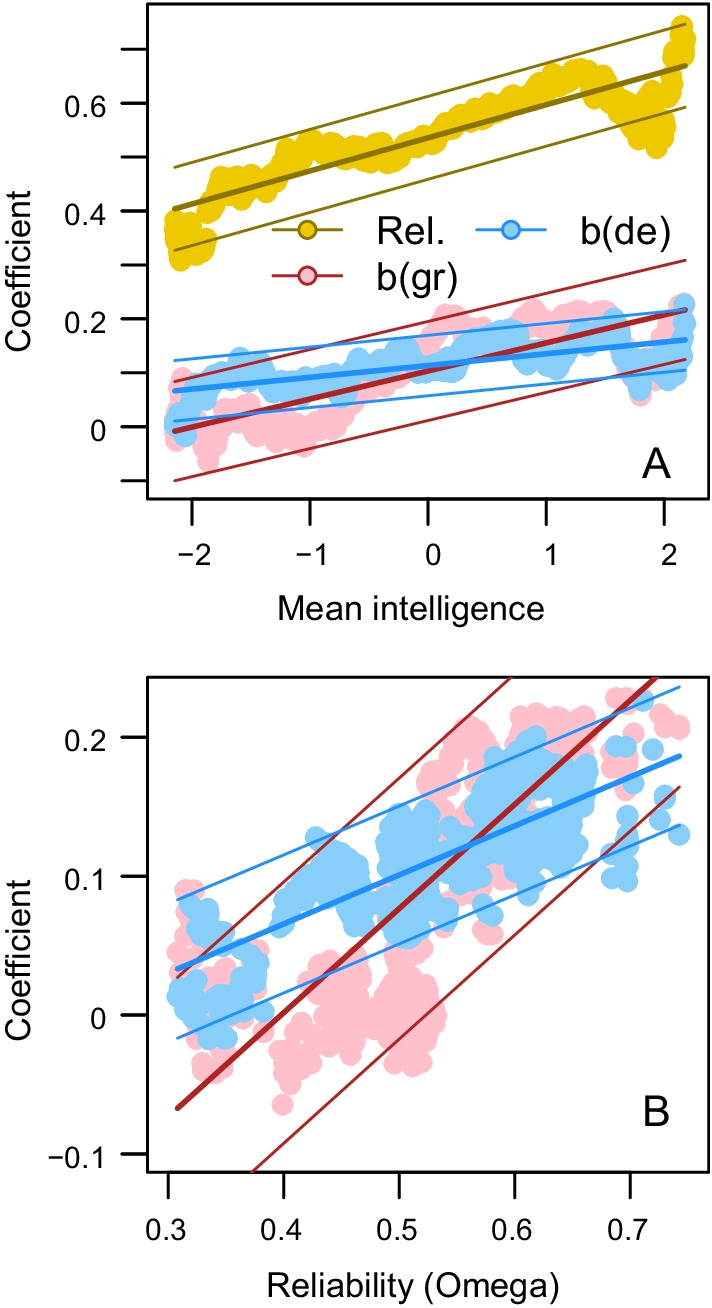
Reliability (Rel., yellow), as well as effects of the dependable composite on grades (b(gr), red) and on degree (b(de), blue), as functions of the mean intelligence (**A**) and of the reliability in the measurement of the dependable composite (**B**) in subgroups. The thinner solid lines indicate the 95% prediction interval

In accordance with the findings above, mean intelligence in the subgroups had a positive effect on the reliability in the measurement of the dependable composite (*b* = 0.061, *p* < 0.001), as well as on the effects of the dependable composite on grades and degree (see below for coefficients) (Fig. 4, panel A). Additionally, the effects of the dependable composite on grades and degree in the subgroups were positive functions of the reliability in the measurement of the composite (*b* = 0.749, *p* < 0.001 and *b* = 0.353, *p* < 0.001, for grades and degree, respectively, Fig. 4, panel B). If adjusting for the reliability in the measurement of the dependable composite, the effect of mean intelligence in the subgroups on the effect of dependable on grades, i.e. the interaction effect, was attenuated by 42.0% (from *b* = 0.052, 95% CI 0.050; 0.054, to *b* = 0.030, 95% CI 0.025; 0.035). If adjusting for the reliability in the measurement of the dependable composite, the effect of mean intelligence in the subgroups on the effect of dependable on degree was attenuated by 95.2% (from *b* = 0.022, 95% CI 0.020; 0.023, to *b* = 0.001, 95% CI − 0.002; 0.004). As there was no overlap between the confidence intervals of the unadjusted and adjusted effects, the degree of attenuation can be assumed to be significantly higher than zero for both grades and for degree.

It can be noted that the personality traits contribute quite little to the prediction of academic achievement over and above the contribution of intelligence. The linear association with intelligence can account for 20.4% and 26.3% of the variance in grades and in degree, respectively, and these values increase to maximally 25.5% (with the disorganized item) and 27.8% (also with the disorganized item) in full models including quadratic terms as well as a personality trait by intelligence interaction term (as in Eq. 1).

## Discussion

The present findings suggest that some indicators of personality traits, mainly related to dependability, interact with intelligence in their association with academic achievements, mainly high school grades. Furthermore, personality trait by intelligence interaction effects tended to be synergistic, which means that the association between the trait and grades, positive or negative, tended to strengthen with increased intelligence. One possible reason for this is that intelligence may be some kind of necessary condition for academic achievement, and if intelligence is too low it cannot be compensated for by a high degree of some advantageous trait like dependability. On the other hand, if intelligence is low, a high degree of some disadvantageous trait like being very critical or careless may not decrease academic achievement much further, i.e. there may be some kind of floor effect involved. For those with sufficiently high intelligence, on the other hand, personality traits may contribute to pushing grades a little bit up- or downward, although the influence of personality traits seems quite limited compared with intelligence.

Observed associations tend to weaken with a decrease in the reliability of measurements. Consequently, a positive association between intelligence and the reliability in the measurement of personality traits, as observed in the present study, could be another contributing cause to the tendency for synergistic personality trait by intelligence interaction effects on grades. The present findings involving a composite measuring dependability indicate that differences in reliability might account for a substantial portion of synergistic interaction effects on academic achievement, although it is difficult to know to what degree this finding generalizes to other populations and other personality trait composite scores.

Earlier studies have found, for example, positive associations between respondents’ intelligence and response consistency on measures of the Big Five personality traits [15–17], but also on measures of psychological maturity, impulsivity, aggression, and callousness [18], measures of youth delinquency [19], and attitudes toward a strong military defense [20]. It has been proposed that this association between intelligence and reliability in measurements may be due to, for example, lack of motivation/carelessness, worse working memory, inadequate reading ability, and poorer understanding of questionnaire items among some respondents, which would tend to result in low performance on intelligence tests as well as low reliability in measurements of attitudes and personality traits [16, 20]. It has also been suggested that people with high intelligence actually tend to have more distinctive personalities compared to those with lower intelligence [21].

In the present study, personality traits by intelligence interaction effects were mainly seen on grades and to a lesser degree on highest degree achieved. One reason for this difference could be the slightly stronger association between intelligence and degree (β = 0.506) compared to the association between intelligence and grades (β = 0.459). This could result in less room for personality traits to have an influence on degree than on grades when including intelligence as a predictor in the model.

The measurement of personality traits is the most obvious limitation in the present study. We used mostly single item measures and the three composite scores had mediocre reliability/consistency. However, low reliability in measurements should tend to decrease power rather than increase risk for type 1-errors. With more reliable full-scale measures of personality traits, we probably had seen more significant synergistic personality trait by intelligence interaction effects rather than elimination of the presently observed interactions or, even less likely, a reversal from synergistic to compensatory interaction effects.

Another limitation is that the personality trait items were measured in 2002 and 2008, when the respondents were between 18 and 22 and between 24 and 28 years old, respectively, i.e. in many cases after they had received their high school grades and achieved their highest academic degree. Therefore, any strong conclusions about causality are excluded and there is a possibility that academic achievement may have influenced the respondents’ self-view and, consequently, how they rated themselves on the personality trait items. Studies have indicated an association between changes in students’ investment in achievement behavior and changes in their personality traits, especially conscientiousness [22]. Furthermore, young adults on a more vocationally oriented path seem to show a larger increase in conscientiousness while individuals attending university show a larger increase in agreeableness [23].

Moreover, it is possible that a reversed causal effect of academic achievement on self-rated personality traits may tend to be stronger among individuals with high compared to those with low intelligence. The self-view of individuals with low intelligence may, as a manner of speaking, be more immune to actual outcomes. This could possibly explain, at least to some degree, the synergistic personality trait by intelligence interaction effects on academic achievement observed in the present and some other studies. Whether or not intelligence moderates a possible effect of outcomes, academic or otherwise, on self-view could actually be an interesting subject for future research, with the added benefit that outcomes, differently from self-rated personality traits, can be experimentally manipulated.

## Conclusions

Personality trait by intelligence interaction effects on grades tend to be synergistic, i.e. the association between the trait and grades, if it exists, tends to strengthen with increased intelligence. This could, for example, be due to a sufficient level of intelligence being necessary for higher grades, and that lack thereof cannot be compensated for by a high degree of some advantageous personality trait. A positive association between intelligence and reliability in the measurement of personality traits, as well as a reversed causal effect of grades on self-rated personality traits moderated by intelligence, could also be contributing causes to the tendency for synergistic interaction effects. Analyses in the present study indicated that differences in reliability between those with high and low intelligence have the potential to account for a big portion of some synergistic personality trait by intelligence interaction effects on academic achievement.

## Data Availability

The script and data are available at Open Science Framework at https://osf.io/2fsca/
